# Multi-institutional perspectives on team science evaluation in clinical and translational science programs: Practices, challenges, facilitators, and future directions

**DOI:** 10.1017/cts.2026.10732

**Published:** 2026-03-31

**Authors:** Whitney A. Sweeney, Joe Hunt, Tammy J. Sajdyk, Boris Volkov

**Affiliations:** 1 Institute for Clinical and Translational Research, School of Medicine and Public Health, University of Wisconsin-Madison, Madison, WI, USA; 2 Indiana Clinical and Translational Sciences Institute, Indianapolis, IN, USA; 3 Indiana University School of Medicine, Indianapolis, IN, USA; 4 Clinical and Translational Science Institute and Institute for Health Informatics, University of Minnesota, Minneapolis, MN, USA

**Keywords:** team science, evaluation, clinical and translational science, CTSA programs, interdisciplinary collaboration

## Abstract

Team science is central to clinical and translational research; however, systematic evaluation of collaborative efforts remains inconsistent and underdeveloped. To better understand current team science evaluation practices within clinical and translational science programs, we conducted a structured cross-sectional survey of team science and evaluation professionals. We analyzed quantitative data using descriptive statistics and qualitative responses through thematic analysis. Among participating organizations, the majority have implemented team science evaluations, predominantly using mixed-methods approaches combining quantitative metrics and qualitative assessments. Evaluation findings were primarily used to inform programing, improve team functioning, and secure funding. Reported challenges fell into four key areas: methodology; implementation; data analysis; and organization. Facilitators included: methodological enhancements, organizational support, collaborative approaches, and infrastructure elements. Participants emphasized using interim measures (e.g., team dynamics and satisfaction) that move beyond traditional outcome measures so that evaluations better reflect how teams interact, adapt, and progress as they develop. While team science evaluation adoption is substantial among leading translational research institutions, significant methodological gaps persist. Future directions focus on developing standardized frameworks with local flexibility, creating validated instruments, utilizing interim process measures, and demonstrating return on investment (ROI) to advance both evaluation science and translational outcomes.

## Introduction

The increasing complexity of biomedical challenges has driven a fundamental shift toward collaborative approaches in clinical and translational research [1,2]. Team science, defined as collaborative research conducted by interdependent investigators across disciplinary boundaries, has become essential for addressing multifaceted problems requiring broad expertise [3]. The Clinical and Translational Science Awards (CTSA) program exemplifies this collaborative imperative, with team science established as a strategic priority by the National Center for Advancing Translational Sciences (NCATS) [4]. An example of NCATS commitment was the Great CTSA Team Science Contest which received 170 submissions from 45 unique CTSA hubs, revealing “a great variety of team science strategies for virtually all team science stakeholders.” [5] This demonstrated the widespread adoption of collaborative approaches across the consortium.

Within the CTSA context specifically, team science addresses unique research challenges that differ from traditional disciplinary approaches. The CTSA program’s emphasis on bridging basic science to clinical application creates distinctive demands for collaborative teams that must integrate different methodological approaches, institutional contexts, and stakeholder perspectives [6]. Although team science is widely adopted across disciplines, translational research requires evaluation methods that assess both standard research productivity and CTSA-relevant translational outcomes. Team science within this context generates unique processes (e.g., cross-institutional collaboration mechanisms and translational workflows), distinct outcomes (e.g., spanning bibliometrics to community health improvements), and specific impacts (e.g., career development in team-based research and institutional capacity building). Understanding evaluation practices within this context is critical for advancing both team science effectiveness and translational research outcomes.

Despite widespread adoption of team science, systematic evaluation of collaborative research efforts remains inconsistent and methodologically underdeveloped [7]. This gap creates significant challenges for program improvement, resource allocation, and demonstration of collaborative research value. While traditional academic metrics adequately assess individual scholarly productivity, they fail to capture the unique processes, outcomes, and impacts generated through team-based research. Evaluation efforts have historically overemphasized objective, outcome-focused measures (e.g., bibliometrics) while underemphasizing assessment of team functioning (e.g., team member satisfaction, trust, and resilience) [6,8,9].

The National Academies report “Enhancing the Effectiveness of Team Science” highlighted evaluation as essential for improving collaborative research processes [2]. More recently, the 2025 National Academies report “The Science and Practice of Team Science” emphasized that systematic evaluation supported by both institutions and team leaders strengthens individual teams and advances team science research [7]. The report identified critical evaluation domains including team social processes, performance outputs, and individual member impacts.

NCATS endorses a systems approach that uses organizational policies, structures, and processes to reinforce the value of team science and enable collaborative environments [4]. Performance review criteria within this framework include not only traditional measures of productivity and impact but also novel measures of effective cross-disciplinary team science (e.g., successful interdisciplinary communication and mutual learning) and contributions to translation (e.g., community partnerships and policy influence). This is critical for individuals on translational teams, which operate at the intersection of knowledge generation and product development, requiring evaluation that captures both discovery-oriented and implementation-focused activities [6]. This comprehensive approach acknowledges that translational outcomes extend beyond conventional metrics and require expanded frameworks. Additionally, other recent frameworks have emphasized evaluating both proximal outcomes (e.g., team processes, member satisfaction) and distal outcomes (e.g., scientific impact, translational applications) across multiple levels of analysis, recognizing that team science unfolds over time [7,10,11].

This study addresses critical gaps in understanding current team science evaluation practices within translational research settings by documenting the evaluation landscape among leading translational research institutions. We sought to: (1) characterize current evaluation practices among CTSAs; (2) understand how evaluation findings are applied; (3) identify evaluation challenges and describe factors that enhance evaluation capability; (4) describe measures of team success; and (5) explore future directions for advancing translational team science evaluation through collaborative initiatives and identify emerging opportunities for field-wide advancement.

## Methodology and data analysis

### Survey design and participant recruitment

We conducted a cross-sectional survey of team science and evaluation professionals within translational research institutions. Participants were recruited through the Association for Clinical and Translational Science (ACTS) Special Interest Groups (SIGs), particularly the Evaluation and Team Science Professionals (TSP) SIGs. The survey was distributed electronically in April–May 2025. This project was deemed program evaluation and exempt by the IRB.

Recruitment was conducted via electronic mail through ACTS SIGs, reaching approximately 350–400 individuals across the two SIGs. Based on estimated SIG membership, the response rate was approximately 10%–15% with nearly one third of CTSAs represented.

The structured questionnaire assessed multiple domains: organizational demographics, team science experience, evaluation practices and methods, implementation challenges, enhancement factors, important outcomes and metrics, and collaborative priorities. Questions employed multiple-choice and open-ended response formats to capture the breadth and depth of evaluation experiences. The instrument was pilot-tested with evaluation and team science experts and refined based on feedback. See Supplementary Materials for specific survey items.

### Data analysis

Quantitative data were analyzed using descriptive statistics, with frequencies and percentages calculated for categorical variables. Qualitative responses were analyzed using thematic analysis, with investigators independently coding responses and resolving discrepancies through consensus. Themes were organized into conceptual categories juxtaposed with the existing team science evaluation knowledge.

## Results

### Participant characteristics

Thirty-five (*n* = 35) team science and evaluation professionals completed the survey with 91.4% affiliated with CTSA institutions, 5.7% (*n* = 2) with Clinical and Translational Research institutions, and 2.9% (*n* = 1) with government organizations. The majority (88.6%, *n* = 31) belonged to ACTS SIGs. The Evaluation SIG was most popular (60.0%, *n* = 21), followed by the TSP SIG (45.7%, *n* = 16). In terms of team science experience, 11.4% (*n* = 4) had <1 year of experience, 47.5% (*n* = 17) had 1–5 years of experience, and 42.8% (*n* = 15) participants had 6 or more years of experience. TSP SIG members tended to be more experienced, with 31.3% (*n* = 5) having >10 years compared to 25.7% overall. Professional roles encompassed five primary categories: evaluation-focused (e.g., program evaluators, directors of evaluation), team science-focused (e.g., team science leads, core scientists), leadership roles (e.g., program directors, associate directors), workforce development (e.g., education directors, training coordinators), and administrative roles (e.g., program administrators, senior staff).

### Current evaluation practices

Among survey respondents’ organizations, 68.6% (*n* = 24) have implemented team science evaluations, while 20.0% (*n* = 7) have not conducted evaluations and 11.4% (*n* = 4) were uncertain about evaluation status. Among the CTSA institutions represented, the evaluation adoption rate was 75.0%.

**Figure 1.**
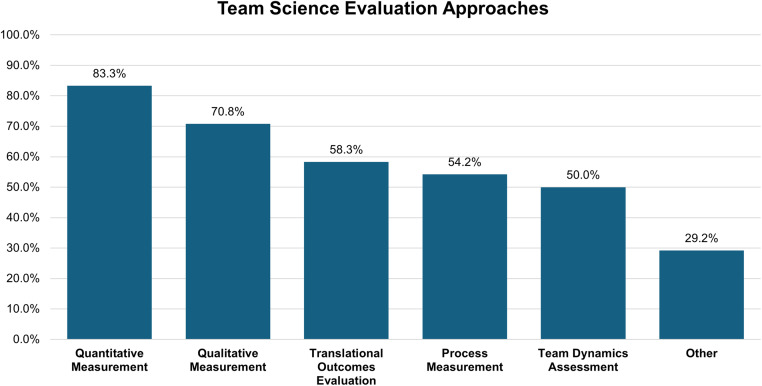
Team science evaluation approaches across clinical and translational science programs. This sample includes the 24 respondents who indicated that team science evaluation was conducted at their institution.

The 24 respondents reporting conducting team science evaluations employed varied approaches, with mixed-methods strategies predominating. Quantitative measurement was most common (83.3%, *n* = 20), focusing on publications, grants, and citations. Qualitative approaches followed closely (70.8%, *n* = 17), incorporating interviews and case studies. Process measurement (e.g., meeting frequency, role clarity) was used by 54.2% (*n* = 13), team dynamics assessment (e.g., trust, communication) by 50.0% (*n* = 12), and translational outcomes (e.g., clinical/community impact) evaluation by 58.3% (*n* = 14). Organizations tended to use 3 evaluation methods on average. The use of multiple complementary methods reflects a growing sophistication in evaluation approach, moving beyond single-method limitations to leverage the strengths of both quantitative and qualitative data (see Figure 1).

Evaluations of team science were conducted at varying times and frequencies, reflecting an integration of assessment into routine organizational processes: 54.2% (*n* = 13) conducted annual assessments, 54.2% (*n* = 13) evaluated at project milestones, and 37.5% (*n* = 9) performed pre/post funding evaluations. This variation in evaluation timing reflects organizational differences in how teams are organized and how evaluation can be most meaningfully integrated into research processes.

### Application of evaluation findings

**Figure 2.**
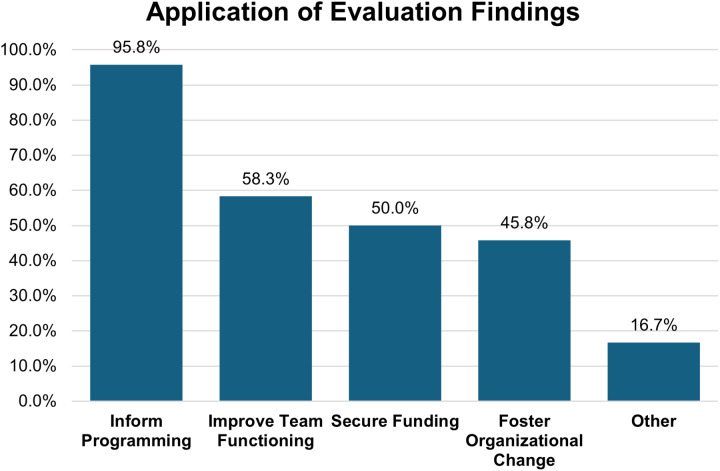
Application of evaluation findings across clinical and translational science programs. This sample includes the 24 respondents who indicated that team science evaluation was conducted at their institution.

Evaluation findings served multiple strategic purposes. Nearly universal application occurred for informing programing (95.8%, *n* = 23), demonstrating practical utility for continuous improvement. Other applications included improving team functioning (58.3%, *n* = 14), securing funding (50.0%, *n* = 12), and fostering organizational change (45.8%, *n* = 11). Respondents reported applying evaluation findings for approximately 3 purposes, indicating broad utilization of evaluation results (see Figure 2).

### Evaluation challenges

Survey participants identified challenges that can be organized into 4 key challenge domains: methodological, implementation, data and analysis, and organizational.

**Methodological challenges** arise from difficulties defining, measuring, and comparing key concepts across contexts. A central issue is *operationalization.* As one respondent emphasized, “One of the biggest challenges is in operationalizing our constructs. What is team science? How do we define impact?” Without shared definitions, evaluations vary widely. These challenges are compounded by a *lack of validated instruments*. *Standardization* is also difficult, since methods that work in one organizational setting often do not translate to others, limiting cross-team or cross-institutional comparisons. *Attributing outcomes to team science initiatives* is hard in complex systems, where isolating effects of a single intervention is rarely possible. These issues complicate rigorous evaluation, limit generalizability, and hinder building an evidence base about what works, for whom, and under what conditions.

**Implementation challenges** reflect the realities of conducting team science evaluations in real-world settings and can significantly affect data quality and interpretability. *Resistance to evaluation efforts* may limit participation and buy-in, while *low survey response rates* threaten representativeness. *Time constraints faced by both evaluators and participants* delay data collection and limit engagement, and *team member turnover complicates longitudinal evaluation*. As one participant explained, “Team science PIs/research members/survey responders often face time constraints, which slow down their response time for evaluation survey or email inquiry and prevents them from completing survey. This can result in low response rate in data collection and missing data.” These challenges constrain feasibility and rigor of evaluations, introduce bias, and make it harder to draw reliable conclusions about team functioning and outcomes over time.

**Data and analysis challenges** highlight structural limitations in collecting, managing, and interpreting evaluation data, weakening conclusions about team science initiatives. *Long-term follow‑up* is often difficult, limiting insight into sustained outcomes. These challenges are compounded by *insufficient data to support comparative analyses* and *small sample sizes that reduce statistical power* and restrict subgroup analyses. *Inconsistent documentation across teams and programs* further undermines data integration and comparability. As one respondent explained, “One of the primary challenges is the lack of sufficient data to conduct comparative or group-based analyses, which limits our ability to assess the true impact of leadership-focused team science training programs. Another challenge involves the longitudinal tracking of program participants, which is essential for understanding how team science training influences their long-term career development.” These challenges limit evaluators’ ability to demonstrate impact, detect differential effects, and build a robust evidence base for understanding how and when team interventions produce meaningful outcomes.

**Organizational challenges** reflect structural, cultural, and resource-related barriers within institutions or networks that limit effective implementation and evaluation of team science. Participants described *varying levels of engagement across networks*, with some teams or leaders less willing to participate in evaluation. *Complex organizational structures* complicate coordination and data flow, making it difficult to track teams, align documentation practices, or aggregate information. *Competing institutional priorities* constrain the time, funding, and attention devoted to evaluation, while *inadequate numbers of dedicated evaluation personnel* limit the capacity to manage and analyze data at scale. As one participant noted, there is sometimes “resistance to submitting and participating in evaluation efforts,” while another highlighted “…inconsistency in the way programs document team members, inconsistent responses from team members, and underutilization of services to support team science.” These challenges weaken evaluation infrastructure, reduce data quality, and impede the ability to systematically assess the effectiveness and value of team science initiatives.

### Evaluation facilitators

Participants were asked to identify specific factors that enhanced their program’s ability to evaluate team science. The four domains that emerged included methodological enhancements, organizational support, collaborative approaches, and infrastructure elements.

**Methodological enhancements** involve improvements to evaluation design and practice that strengthen the rigor, relevance, and usefulness of team science assessment. Participants emphasized *integrating qualitative methods alongside quantitative measures*, noting that interviews, observations, and open‑ended feedback provide richer insight into team dynamics, collaborative processes, and contextual factors. As one respondent stated, “Bringing qualitative methods into the evaluation folder has helped tremendously.” *Setting clear evaluation expectations early* was also identified as critical for aligning around shared goals, roles, and data collection requirements. *Centralized tracking systems* were described as improving consistency, data management, and longitudinal follow‑up. Participants highlighted *meaningful, measurable, and actionable metrics* that accurately reflect team science outcomes and support decision‑making. These enhancements address methodological limitations by increasing measurement validity, strengthening comparability, and enabling more rigorous evaluations.

**Organizational support** factors reflect the structural and cultural conditions that enable effective, integrated, and sustainable evaluation. Participants emphasized that *strong leadership commitment* and dedicated time allocation signals the importance of evaluation and legitimizes the effort. *Broader institutional backing* strengthens evaluation capacity, and PIs are more likely to support evaluation when they perceive *clear benefits* (e.g., improved productivity or competitiveness for funding). When these supports are in place, *evaluation is more easily embedded into routine organizational processes*. As one participant noted, “Setting evaluation expectations at the beginning of a project, getting buy-in from teams” enhanced their program’s ability to evaluate team science within their CTSA. These support factors promote consistent participation, improve data quality, and help make team science evaluation a standard and enduring component of institutional practice.

**Collaborative approaches** focus on strengthening interpersonal and interprofessional relationships to enhance evaluation capacity. Participants emphasized *strong partnerships between evaluators and team scientists*, noted that close collaboration ensured evaluations are relevant, methodologically sound, and aligned with scientific goals. *Working across disciplines and roles* allows teams to leverage unique perspectives enriching evaluation design and supporting nuanced interpretations. *Knowledge sharing around team science and evaluation theories and practices* builds collective expertise, improving both rigor and applicability. Respondents highlighted the importance of cultivating specialized expertise, with individuals and teams *developing advanced skills at the intersection of evaluation and team science*, enabling more sophisticated and context-sensitive assessments. As one participant explained, “We have a fabulous evaluation team! We enjoy strong collaborations between the eval team and the team science core. Thus, we can bring their knowledge of metrics and their infrastructure for regular assessment and combine it with our knowledge about how teams work and the factors that impact their work.” These approaches promote shared ownership, strengthen methodological quality, and enhance the usefulness of findings for team science.

**Infrastructure elements** include the technical and logistical systems that enable efficient, high-quality evaluation of team science initiatives. Participants valued *streamlined data management* platforms like REDCap, which support secure, consistent metric tracking. *Dedicated personnel who assist with data collection and management* were essential for reducing burden and improving data accuracy. *Centralized activity monitoring* was highlighted, as consolidating information allows organizations to identify patterns, detect gaps, and report outcomes more efficiently. *Effective communication systems* were critical for showing the value of evaluation and fostering stakeholder trust. As one participant noted, “Team science project managers work closely [with their teams] to increase awareness of various evaluation needs and deadlines,” enhancing evaluation efforts within their CTSA. These elements strengthen evaluation capacity by improving coordination, data quality, and transparency.

### Measures of success

Participants identified four outcome domains, each with metrics they viewed as most important for evaluating team science performance and growth.

**Process measures** assess the quality and dynamics of team interactions and how collaborative work is carried out. Participants described both *broad assessments of the collaborative experience*, often framed as “teaming,” and more nuanced measures of team functioning. These include measures of *satisfaction, trust, and leadership skill development*, which capture how effectively teams communicate, build relationships, and coordinate their work. Together, process measures provide insight into the social and relational mechanisms that underlie successful team science.

**Productivity measures** capture quantifiable outputs that reflect the effectiveness and accomplishments of collaborative research. Survey responses emphasized traditional metrics, *publications*, *grant funding*, and *project completion rates*, alongside measures of *cross-institutional collaboration that reflect the breadth and depth of partnerships*. Participants also described the value of *documenting collaborative track records* that synthesize quantitative outputs with qualitative information about individual contributions, team processes, and broader scholarly influence. These measures demonstrate the tangible results of team-based research while contextualizing how those results are achieved.

**Impact measures** assess the broader effects and long-term value beyond immediate research outputs. Participants highlighted outcomes related to *career trajectories*, including advancement, leadership roles, and expanded research opportunities, as well as *community influence* and engagement. Measures of *translation to practice* and real-world implementation were also emphasized, aligning with the *Translational Science Benefits Model (TSBM).* These measures capture health, economic, policy, and social benefits, offering a more comprehensive view of the sustained and societal value of team science initiatives.

**Learning measures** focus on growth in knowledge, skills, and capacity among individuals and teams engaged in collaborative research. Participants described assessing *changes in individual awareness, knowledge, skills, and behaviors*, as well as *shifts in team-level practices* such as communication and conflict resolution. These measures show how engagement in team science fosters learning at multiple levels, supporting continuous improvement, and strengthening teams’ ability to collaborate effectively over time.

### Future directions for evaluating team science

When asked for recommendations on evaluating team science within translational science, participants identified several future directions across four key domains.

**Methodological advancements** reflect forward-looking improvements in the execution of team science evaluation that enhance rigor, comparability, and scalability. Respondents highlighted standardized approaches that enable *cross‑institutional comparison while remaining adaptable to local contexts*. *Validated tools for longitudinal tracking* help capture team development and sustained outcomes, while rigorous designs such *as randomized trials or multiphase optimization strategies* can strengthen causal inference. Participants stressed the value of *large‑scale, coordinated data collection* to support generalizable evidence bases. These advancements signal a shift toward evaluation frameworks that, “move beyond outputs to include process and real‑world impact, by fostering continuous learning, system‑wide improvement, and better health outcomes.”

**Conceptual development advances** strengthen the theoretical foundations guiding team science evaluation. Participants valued *flexible models that account for stages of team development*, since evaluation priorities and metrics shift across the team lifecycle. *Competency‑based assessment frameworks* help to identify core capacities, and respondents called for *dynamic measures that move beyond binary classifications* to capture changes over time. A deeper *understanding of how teams evolve* across project lifecycles was seen as essential. As one participant noted, “We need to develop flexible models based on team development stages and different translational starting points as well as flexibility in outcome criteria (since different teams have different anticipated outcomes).” These advances support nuanced, context‑sensitive evaluations that reflect the complexity and developmental nature of team science.

**Measurement innovation** focuses on developing refined metrics that enable timely, meaningful evaluation of team science, particularly in complex and rapidly evolving environments. Participants emphasized the need for *interim measures of team function* that capture team development without multi‑year follow‑up and for approaches that *demonstrate return on investment (ROI)* for team initiatives. *Interdisciplinary evaluation strategies* were seen as essential for honoring varied research paradigms, while *participatory evaluation methods* served as a way to improve relevance, interpretation, and uptake of findings. As one respondent articulated, “Moving beyond achievements and binary competencies (met/unmet) to something more dynamic and locally‑relevant to that particular context… teams are hard to shoehorn into bounded sets with predictable and observable characteristics.” These innovations reflect a shift toward evaluation approaches that balance standardization with contextual sensitivity, capturing team development in rigorous and meaningful ways.

**Impact focus** ensures that team science is productive and aligned with translational goals that improve health and well‑being. Respondents highlighted *measuring cost‑effectiveness* and resource efficiency alongside indicators of *process quality, community benefit, and continuous learning outcomes*. Rather than viewing impact as static, participants emphasized understanding how teams adapt as projects evolve or change in response to new evidence or context. As one respondent explained, “The future directions for evaluating team science, in my opinion, is understanding how teams change team members as their projects move along the translational spectrum or change direction completely. I think understanding how quickly or what barriers exist for teams as they need to adapt to change may be a marker for understanding success.” This impact-focused lens reframes evaluation as a tool for capturing adaptability and real‑world value, key indicators of meaningful and sustainable team science outcomes. Table 1 summarizes the key conceptual categories and examples derived from the qualitative analysis.

**Table 1.** Summary of the insights from the multi-institutional survey on team science evaluation challenges, facilitators, measures, and future directions

Summary of insights from the multi-institutional survey	
Evaluation challenges	
Methodological	Operationalizing constructs Lack of validated instruments Standardization of methods/metrics across contexts, and Attribution of causality to team science interventions
Implementation	Resistance to evaluation efforts Low survey response rates Time constraints for both evaluators and participants Team member turnover complicating longitudinal data collection
Data and analysis	Long-term follow-up Insufficient data for comparison Small sample sizes limiting statistical power Inconsistent documentation across teams and programs
Organizational	Varying engagement levels across networks Complex organizational structures Competing priorities limiting evaluation resources Inadequate dedicated evaluation personnel
Evaluation facilitators	
Methodological enhancements	Complementing quantitative data with qualitative methods Establishing evaluation expectations early Using centralized tracking systems Developing meaningful, measurable and actionable metrics
Organizational support	Strong leadership commitment Obvious clear benefits Broad institutional backing Integration of evaluation into organizational processes.
Collaborative approaches	Strong partnerships between evaluators and team scientists Integration of unique perspectives across roles and disciplines Team science and evaluation knowledge exchange Advanced expertise development
Infrastructure elements	Streamlined data management Dedicated data collection and management personnel Centralized activity monitoring Evaluation impact messaging
Measures of success	
Process	Broad assessment of collaborative experience (“teaming”) Member satisfaction Team trust Development of leadership skills
Productivity	Publication and grant funding Project completion rates Depth and breadth of cross-institutional collaborations Individual collaborative track records
Impact	Career trajectory impacts Community influence Translation to practice Benefits suggested by Translational Science Benefits Model (TSBM)
Learning	Changes in individual awareness, knowledge, skills, and behavior Changes in team-level skills or practice
Future directions for evaluating team science	
Methodological advancements	Standardized cross-institutional approaches that maintain local adaptability Validated tools for longitudinal tracking of team progress and outcomes, Randomized trials or multiphase optimization strategies Large-scale coordinated data collection
Conceptual development advances	Flexible models based on team development stages Competency-based assessment frameworks Dynamic measures that move beyond binary competencies Understanding team change and adaptation
Measurement innovation	Developing interim measures of team function Demonstrating return on investment (ROI) Interdisciplinary evaluation approaches Participatory evaluation methods
Impact focus	Cost-effectiveness Process quality Community benefit Continuous learning outcomes

## Discussion

This study documents the current state of team science evaluation practices among leading clinical and translational science institutions. The findings reveal a landscape characterized by substantial adoption of evaluation activities and increasing methodological sophistication. The predominance of mixed-methods strategies reflects growing recognition that team science requires multifaceted measurement approaches [7]. Greater emphasis on process and outcome evaluation reflects best practices showing that team functioning and processes predict downstream research outcomes [7,10].

The substantial attention to translational outcomes (58.3%) indicates alignment with CTSA program objectives [12] and TSBM framework principles [11,13]. This represents an important shift toward valuing the full translational pipeline beyond traditional academic metrics. The finding that 96% of organizations conducting evaluation use findings to inform programing demonstrates evaluation’s strategic integration into continuous improvement processes, aligning with recommendations that evaluation should support both accountability and enhancement functions [14]. Team science evaluation was also used to foster organizational change (45.8%) and secure funding (50.0%), further underscoring their strategic utility. However, close to a third of organizations have not implemented evaluation of team science. This finding warrants careful interpretation. It does not necessarily indicate a lack of team science activities at these organizations; rather, it may reflect challenges in systematically evaluating existing team science initiatives, resource constraints limiting evaluation capacity, or differing organizational priorities regarding evaluation. Future research should clarify whether evaluation gaps reflect true absence of team science programs or implementation barriers that prevent evaluation of existing programs.

The identification of construct operationalization as a primary challenge highlights a fundamental issue noted in the National Academies report: the need for clear, standardized definitions of team science and related constructs [7]. Without consensus on what we mean by key terms, consistent measurement becomes difficult. The lack of validated instruments represents a critical gap requiring coordinated field-level attention. As multiple respondents noted, validated measures, evidence-based approaches to evaluations, and standardized methods of implementation are essential for advancement. However, these cannot be developed in the absence of clear construct definitions. We therefore recommend a phased approach: first, establish consensus definitions through expert working groups; second, develop instruments based on these definitions; and third, validate instruments through multi-site testing.

The implementation challenges align closely with those documented in the broader team science literature, including methodological complexity, resource constraints, and participant burden [1]. The prominence of low response rates and time constraints reflects broader challenges in evaluating complex collaborative processes while minimizing disruption to scientific productivity. These challenges underscore the importance of early integration of evaluation expectations, adequate resourcing, and explicit communication of evaluation value to team members.

Enhancement factors (i.e., facilitators) provide a roadmap for addressing these challenges. The emphasis on early evaluation integration, leadership commitment, and cross-functional collaboration suggests that successful evaluation requires systematic organizational investment rather than ad hoc implementation. The success of centralized tracking systems and dedicated evaluation expertise indicates the value of infrastructure investment for sustainable evaluation programs.

These findings contribute to the theoretical understanding of team science evaluation by identifying specific challenges and solutions within translational research contexts. The emphasis on interim process measures extends beyond traditional outcome-focused approaches. This supports dynamic frameworks that account for team evolution and adaptation, allowing organizations to assess team functioning and make improvements throughout the research process rather than only at project completion [7].

The strong interest in cross-SIG collaboration (80%) suggests significant opportunity for field-level coordination that could address standardization challenges while respecting institutional differences and context. The convergence of evaluation and team science expertise within the CTSA consortium, evidenced by cross-functional collaboration and shared learning interests, positions the translational science community to realize the full potential of evidence-based team science evaluation.

### Novel contributions and limitations of this study

This study advances team science evaluation in translational science by: (1) documenting current evaluation practices across leading translational research institutions; (2) identifying how CTSAs apply team science evaluation findings; (3) highlighting key challenges and facilitators based on professional experience; (4) revealing emerging consensus on evaluation purposes, methods, and priorities; and (5) outlining future directions for strengthening translational team science evaluation through collaborative initiatives and field‑wide opportunities. While prior work has emphasized the importance of evaluating team science, this study offers specific examples of how organizations conduct evaluation in practice and where resources may be directed for greatest impact. This study has several limitations. The sample size (*n* = 35) limits generalizability, though it reflects substantial expertise within the CTSA consortium and broader translational research community. The sampling frame was drawn from ACTS Evaluation and TSPs ensured participation from evaluation and TSP but may exclude others involved in team science evaluation within CTSAs or related institutions. The estimated response rate (10–15%) is typical for professional surveys but may introduce non‑response bias toward individuals more engaged in evaluation and team science activities.

The cross-sectional design limits the ability to assess evaluation effectiveness over time; longitudinal studies would better capture how practices evolve and relate to team science outcomes. Self-reported data may introduce social desirability bias, particularly regarding evaluation implementation and success. Because the survey was conducted in April–May 2025, it may not reflect more recent developments. Perspectives were drawn primarily from evaluation and TSPs with quantitative scientists, statisticians, epidemiologists, and informaticians underrepresented potentially limiting insight into evaluation methodologies across all relevant disciplines. However, the convergence of participant responses and alignment with existing literature suggest that findings capture authentic experiences and represent meaningful patterns in current practice.

## Conclusions

Based on the insights provided by team science and evaluation professionals, we propose several recommendations to advance the systematic evaluation of team science within the context of translational science.

### For individual organizations

- Adopt mixed-methods evaluation approaches incorporating both process and outcome measures;
- Establish evaluation expectations early in team formation;
- Invest in dedicated evaluation expertise and infrastructure;
- Implement feedback and communication systems demonstrating evaluation value to participants;
- Use evaluation findings iteratively to inform continuous improvement.

### For the field

- Establish expert working groups to develop consensus definitions for key team science constructs;
- Develop and validate standardized instruments adaptable to different institutional contexts;
- Create shared evaluation frameworks balancing consistency with flexibility;
- Design interim measures complementing long-term impact assessment;
- Foster cross-institutional collaboration on evaluation methodology research.

### For funding agencies

- Require collaboration plans and evaluation components in team science proposals;
- Support evaluation methodology research and instrument validation studies;
- Facilitate data sharing and collaborative evaluation initiatives across institutions;
- Provide resources for evaluation capacity building and professional development.

### For cross-institutional/group collaboration

- Establish working groups addressing specific methodological challenges;
- Develop interoperable data platforms enabling pooled analysis across institutions;
- Create implementation guides and training materials for team science evaluation;
- Design multi-site studies demonstrating team science value and effectiveness;
- Include varied disciplinary perspectives including quantitative scientists, statisticians, epidemiologists, and informaticians in future collaborative work.

### For future research

- Conduct longitudinal studies examining evaluation effectiveness and association with team science outcomes;
- Perform comparative analyses of different methodological approaches;
- Develop and validate standardized instruments;
- Investigate relationship between evaluation practices and team science outcomes.

This study documents progress in team science evaluation within translational programs, with high adoption rates and methodological sophistication among leading institutions. The finding that nearly all evaluating organizations use results for program improvement demonstrates evaluation’s strategic value beyond mere accountability. The convergence of evaluation and team science expertise positions translational programs to realize the potential of evidence‑based evaluation while advancing collaborative research effectiveness to improve human health outcomes

## Supporting information

10.1017/cts.2026.10732.sm001 Sweeney et al. supplementary material Sweeney et al. supplementary material

## Supplementary material

The supplementary material for this article can be found at https://doi.org/10.1017/cts.2026.10732.
