# Supplementary material for: Multi-institutional perspectives on team science evaluation in clinical and translational science programs: Practices, challenges, facilitators, and future directions
Source: J Clin Transl Sci. 2026 Mar 31;10(1):e78. doi: 10.1017/cts.2026.10732 (PMC13184649; doi:10.1017/cts.2026.10732)
Supplement: Sweeney et al. supplementary material [file S2059866126107328sup001.docx]

# Evaluating Team Science in the Context of Translational Science

Translational Science is the process of turning scientific discoveries made in the laboratory, clinic, or community into practical applications to improve human health. It is an interdisciplinary field that involves collaboration among scientists, clinicians, and other stakeholders to ensure that scientific advancements lead to tangible health benefits.

Team Science refers to collaborative efforts involving multiple scientific stakeholders who work together to address a problem, often by integrating diverse perspectives and approaches (Stokols et al. 2008). While much is known about how these interdisciplinary teams function, there is inconsistency in how their evaluation is implemented and clear guidance for evaluating their performance and impact is lacking.

The purpose of this survey is to gain information about the ways in which your organizations evaluate Team Science in the Context of Translational Science, so that we can identify and disseminate helpful strategies, practices, and tools to strengthen evaluation efforts.

Thank you for your valuable input!

(Clinical and Translational Science Award)

What institution or organization do you represent? CTSA CTR

(Clinical and Translational Research Organization)


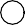

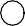

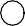


Other

Please describe the institution or organization you represent.

Do you belong to one or more of the ACTS* Special Yes


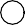

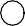


Interest Groups? No

*Association for Clinical and Translational Science

If yes, to which ACTS Special Interest Group (SIG) do you
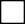
 Team Science Professionals SIG belong? (Check all that apply)
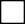
 Evaluation SIG


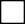
 Scholar SIG


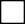
 Biostatistics, Epidemiology, and Research Design SIG


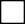
 Clinical Research Professionals Taskforce SIG


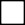
 Informatics SIG


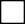
 Translational and Integrative Engagement in Research SIG


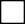
 Partners for the Advancement of Community Engaged Research SIG


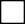
 Training Research Education Administrators in Translational Science SIG


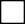
 Research and Operations SIG

Describe your role(s) in your organization (e.g., program evaluator, administrator, researcher, etc.)?

How long have you been involved with team science < 1 year


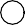

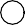

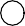

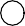


efforts? 1-5 years

6-10 years

>10 years

Team Science in the Context of Translational Science

How does your organization facilitate team science?

What role (if any) do you play in your organization's team science efforts?

Evaluating Team Science

Has your organization/program ever evaluated Yes


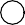

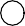

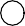


(assessed, measured, rated, etc.) team science No

efforts? Not Sure

If yes, what methods have been used for evaluation?
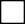
 Quantitative (e.g., publications, grants, (Select all that apply) citations)


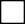
 Qualitative (e.g., interviews, case studies)


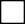
 Process indicators (e.g., meeting frequency, role clarity)


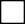
 Team dynamics (e.g., trust, communication)


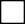
 Translational outcomes (e.g., clinical/community impact)


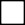
 Other

Please describe

How frequent are your team science evaluations (check
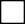
 Pre/post funding all that apply):
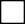
 Annual


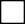
 At project milestones


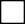
 Not Sure

What challenges have you experienced when evaluating team science?

What specific factors have enhanced your program's ability to evaluate team science?

What else could further enhance your ability to evaluate team science?

What outcomes and associated metrics are most important when evaluating the success of team science efforts?

Please share examples (if any) where team science evaluation helped improve team science (and other translational) processes and outcomes.

How has your organization used evaluation findings?
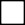
 Inform programming (check all that apply)
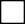
 Secure funding


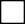
 improve team functioning


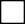
 Foster organizational change


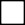
 Other

Please specify

What recommendations would you provide for conducting the evaluation of team science in the context of translational science?

In your opinion, what are the future directions for evaluating team science in the context of translational science?

Would you be interested in participating in a Yes


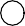

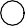


cross-SIG collaboration for evaluating team science? No

If yes, what do you think are the most important questions/issues to address in such a collaboration?

Can we contact you for additional follow-ups (e.g., Yes


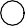

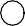


future projects or additional questions)? No

Please provide your name:

Please provide your email address:
